# Identification of hub genes and their correlation with immune infiltrating cells in membranous nephropathy: an integrated bioinformatics analysis

**DOI:** 10.1186/s40001-023-01311-3

**Published:** 2023-11-16

**Authors:** Miaoru Han, Yi Wang, Xiaoyan Huang, Ping Li, Xing Liang, Rongrong Wang, Kun Bao

**Affiliations:** 1 State Key Laboratory of Dampness Syndrome of Chinese Medicine, The Second Affiliated Hospital of Guangzhou University of Chinese Medicine, Guangzhou, China; 2 Guangzhou University of Traditional Chinese Medicine, Guangzhou, China; 3 Guangdong-Hong Kong-Macau Joint Lab On Chinese Medicine and Immune Disease Research, Guangzhou, China; 4 Guangdong Provincial Key Laboratory of Chinese Medicine for Prevention and Treatment of Refractory Chronic Disease, The Second Affiliated Hospital of Guangzhou University of Chinese Medicine, Guangzhou, China; 5 Department of Nephrology, Guangdong Provincial Hospital of Chinese Medicine, Guangzhou, China

**Keywords:** Biomarker, Immune cell infiltration, Least absolute shrinkage and selection operator algorithm, Membranous nephropathy, Weighted gene co-expression network analysis

## Abstract

**Background:**

Membranous nephropathy (MN) is a chronic glomerular disease that leads to nephrotic syndrome in adults. The aim of this study was to identify novel biomarkers and immune-related mechanisms in the progression of MN through an integrated bioinformatics approach.

**Methods:**

The microarray data were downloaded from the Gene Expression Omnibus (GEO) database. The differentially expressed genes (DEGs) between MN and normal samples were identified and analyzed by the Gene Ontology analysis, the Kyoto Encyclopedia of Genes and Genomes analysis and the Gene Set Enrichment Analysis (GSEA) enrichment. Hub The hub genes were screened and identified by the weighted gene co-expression network analysis (WGCNA) and the least absolute shrinkage and selection operator (LASSO) algorithm. The receiver operating characteristic (ROC) curves evaluated the diagnostic value of hub genes. The single-sample GSEA analyzed the infiltration degree of several immune cells and their correlation with the hub genes.

**Results:**

We identified a total of 574 DEGs. The enrichment analysis showed that metabolic and immune-related functions and pathways were significantly enriched. Four co-expression modules were obtained using WGCNA. The candidate signature genes were intersected with DEGs and then subjected to the LASSO analysis, obtaining a total of 6 hub genes. The ROC curves indicated that the hub genes were associated with a high diagnostic value. The CD4^+^ T cells, CD8^+^ T cells and B cells significantly infiltrated in MN samples and correlated with the hub genes.

**Conclusions:**

We identified six hub genes (*ZYX*, *CD151*, *N4BP2L2-IT2*, *TAPBP*, *FRAS1* and *SCARNA9*) as novel biomarkers for MN, providing potential targets for the diagnosis and treatment.

**Supplementary Information:**

The online version contains supplementary material available at 10.1186/s40001-023-01311-3.

## Introduction

Membranous nephropathy (MN) is an immune-mediated glomerular disease characterized by the deposition of immune complexes along the subepithelial region of the glomerular basement membrane [1]. Immunohistochemical staining showed that the deposits contained mainly long-unidentified antigens, IgGs and complement components [2, 3]. The overall incidence of MN is 12 cases per 1 million adults worldwide, of which approximately 80% are idiopathic conditions [4]. Diagnosis is made on kidney biopsy because there is no clear understanding of the molecular mechanisms of the disease. Clarifying the pathogenesis of MN is of importance for clinical diagnosis and treatment.

Immune cells play a relevant role in MN. An imbalance of specific subpopulations of T helper (Th) cells was previously described in the development of MN [5]. Th2 cells release various cytokines that stimulate B cells to produce immune complexes depositing in the glomerular wall. Rituximab, a human-mouse chimeric monoclonal antibody exerting B-cell depleting effects via binding to CD20, is approved for the treatment of MN [6]. Previous studies described an increased proportion of T cell subsets (CD4^+^/CD8^+^) in idiopathic MN, but this finding was not conclusive [7]. The exact nature of the disease-initiating antigen in idiopathic MN remains unknown [8]. Th17 cells are generally regarded as key effectors of autoimmune inflammation, but their role in MN remains unknown [9].

The weighted gene co-expression network analysis (WGCNA) is a widely used bioinformatics method for studying biological networks. The WGCNA identifies hub genes and analyzes potential disease-associated genomic changes [10]. It has been applied to various diseases, such as breast cancer [11], ischemic stroke [12] and rheumatoid arthritis [13]. However, WGCNA has been not used in MN. The least absolute shrinkage and selection operator (LASSO) algorithm is a regression analysis method used for a more accurate prediction in clinical decision making [14].

In this study, we screened and identified candidate biomarkers for MN by combining WGCNA and LASSO analyses. In addition, we used the single-sample Gene Set Enrichment Analysis (ssGSEA) to investigate 28 immune infiltrating cells in MN samples and their correlation with the candidate biomarkers.

## Materials and methods

### Data extraction

The NCBI GEO database (https://www.ncbi.nlm.nih.gov/geo/) was used to get raw files for three registered microarray data sets, GSE200828, GSE108113, and GSE108112 (Table 1) [15]. These datasets were obtained from GPL19983's microarray platform, Affymetrix Human Gene 2.1 ST Array [HuGene21st_Hs_ENTREZG_19.0.0]. Only kidney biopsy from human membranous nephropathy (MN) and kidney biopsy from human healthy living donor (Control) were chosen for each data set. We combined GSE200828 and GSE108113 for our study, and the combined dataset served as the training dataset. The GSE108112 dataset was then used as a test dataset for model validation. The batch effect was removed using the "limma" [16] package's "remove Batch Effect" function [17] in R program. Finally, 181 MN and 16 control samples were included for subsequent analyses. All data were downloaded on 22 October 2022.

**Table 1:** Characteristics of datasets in this study

GSE series	Platform	MN	Control	Total	Submission date
GSE200828	GPL19983	51	6	57	Apr 14, 2022
GSE108113	GPL19983	87	5	92	Dec 14, 2017
GSE108112	GPL19983	43	5	48	Dec 14, 2017

### Identification of differentially expressed genes (DEGs)

We used the “limma” [16] package in R software for the identification and analysis of differentially expressed genes (DEGs) of MN and normal samples in the combined dataset. Filtering was performed based on the false discovery rate (FDR) < 0.05 and log |fold change| (logFC) ≥ 1.5. Volcano plot and heat map of DEGs were plotted using the “ggplot2” [18] and “pheatmap” packages (https://cran.r-project.org/web/packages/pheatmap/index.html).

### Enrichment analysis

We used the “DOSE” [19], “org.Hs.eg.db” [20] and “clusterProfiler” [21] packages for Gene Ontology (GO) and Kyoto Encyclopedia of Genes and Genomes (KEGG) enrichment analyses. The GO analysis includes three components: biological process, molecular function and cellular component. An adjusted *p*-value < 0.05 was used to select significant GO terms and KEGG pathways. The data were visualized using the “ggplot2” [18] package.

### Gene set enrichment analysis (GSEA)

We performed the GSEA to further explore the immune mechanisms in the pathogenesis of MN. The GSEA is used to associate a disease phenotype to a group of genes, attributing a specific weight to each gene in the input list that depends on a metric of choice. Reference genes were downloaded from the Molecular Signatures Database (MSigDB). The adjusted *p*-value < 0.05 was set as the cut-off criterion.

### Weighted gene co-expression network analysis (WGCNA)

We applied the WGCNA method to build a gene co-expression network [10]. Firstly, the samples were clustered to identify any significant outliers. Second, a scale-free network was constructed by calculating the strength of connection between genes. The “pickSoftThreshold” function in the R software was used to calculate the value of β (a soft threshold power parameter), ensuring a scale-free network. Then, a dynamic tree cutting algorithm was used to identify modules by hierarchical clustering. We set the minimum module size to 60 and the cut height to 0.25. Finally, we merged similar modules and evaluated the correlation between module feature genes, clinical features and modules associated with the features. Gene significance (GS) and module affiliation (MM) were calculated for each module and used for hub gene selection. The genes with GS > 0.5 and MM > 0.8 were defined as pivotal genes.

### Identification of the hub genes

We plotted Venn diagrams of key genes and DEGs. The overlapping genes were subjected to the LASSO analysis to identify the hub genes. The LASSO algorithm chooses variables by building a penalty function that reduces the coefficients of non-significant variables to zero while keeping the model correlation coefficients of variables with non-zero regression coefficients [22]. For lasso regression, we utilized the glmnet function, and cross-validation was done with the cv.glmnet function with nfolds = 10. The expression level of the hub genes in MN and normal samples was analyzed using box plots. The receiver operating characteristic (ROC) curves were plotted using the “pROC” [23] package, while the area under the ROC curve (AUC) was calculated to analyze the expression level of the hub genes. We used the GSE108112 dataset and plotted ROC curves to assess the diagnostic value of the hub genes.

### Evaluation of immune cell infiltration and their correlation with the hub genes

The relative infiltration level of 28 immune cells in the combined dataset was quantified using the ssGSEA score [24] and visualized using the "pheatmap" package. Violin plots showed the infiltration level of the immune cells. Spearman correlations between immune infiltrating cells and hub genes were visualized using the “ggplot2” [18] package.

## Results

### Screening and identification of DEGs

**Fig. 1:**
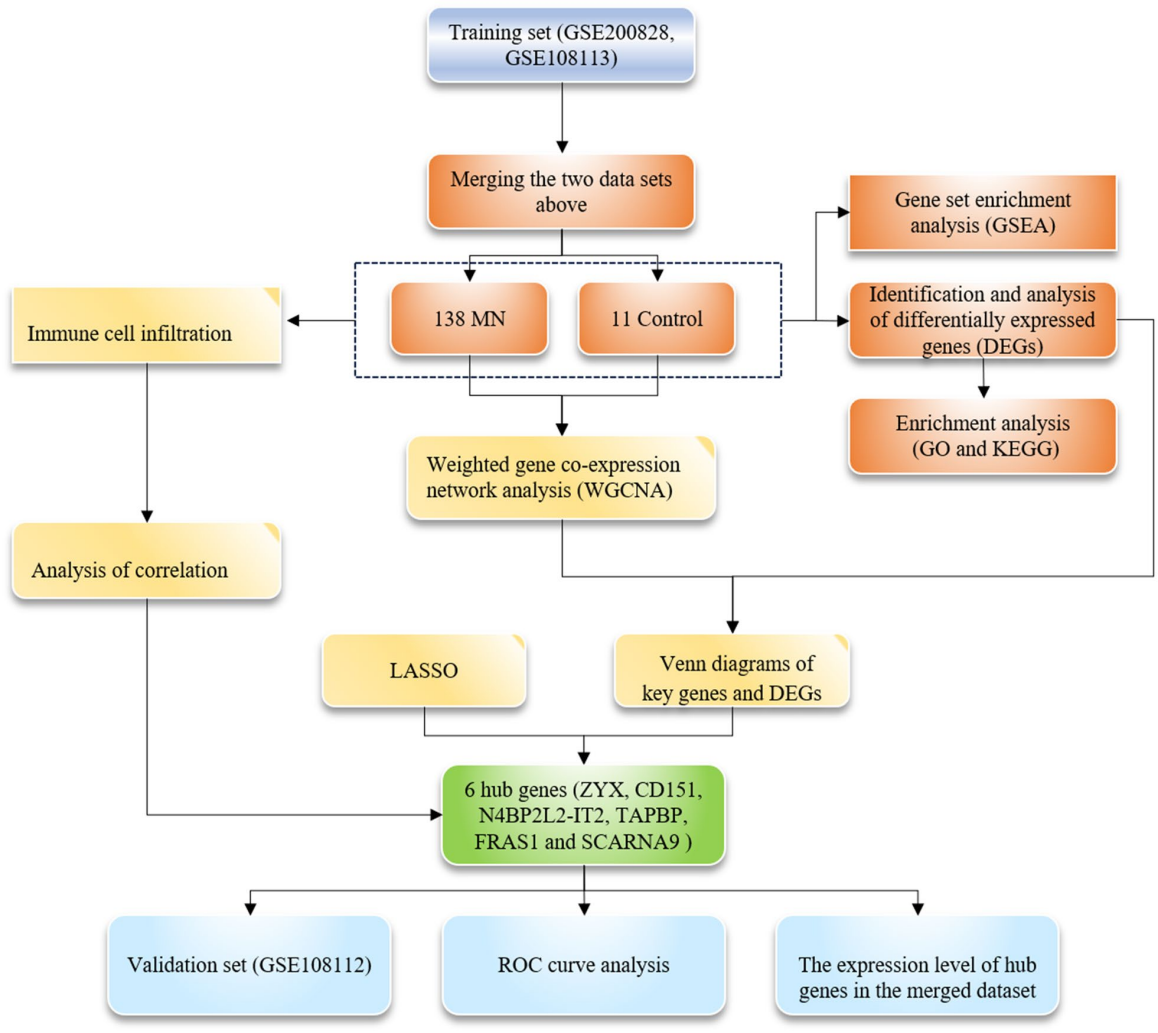
The workflow of the study

**Fig. 2:**
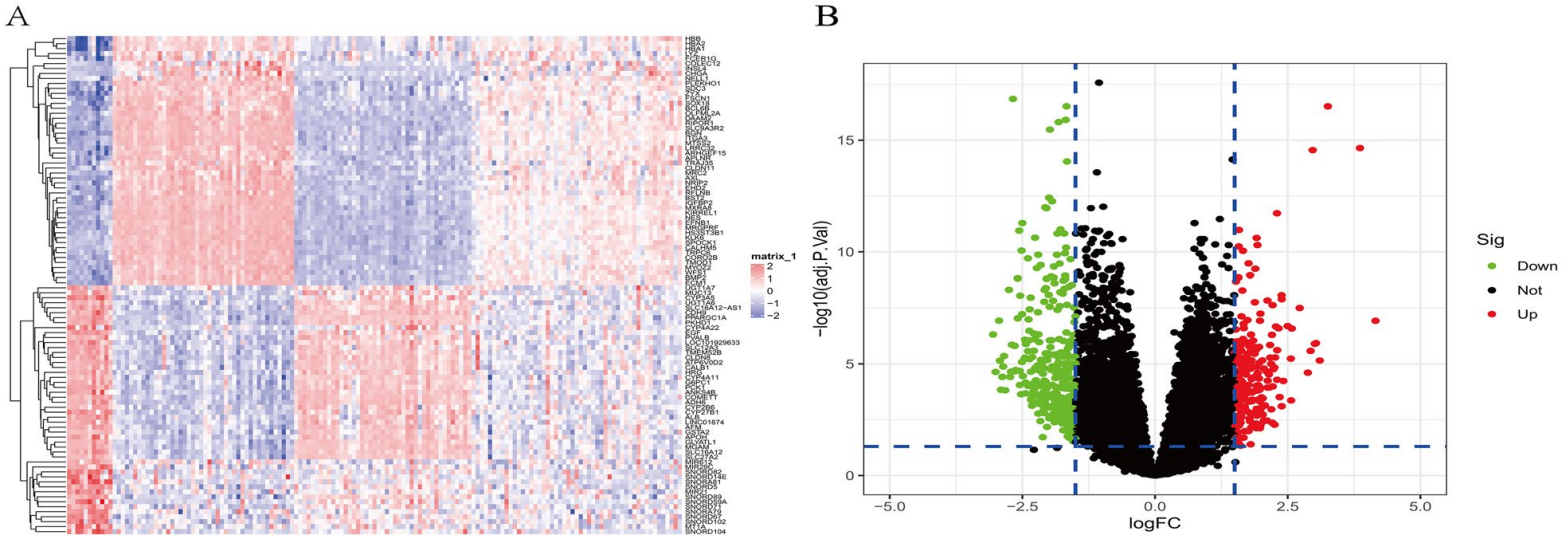
Differential expression analysis of the merged dataset. **A** Heatmap plot of differentially expressed genes for conditions of interest. **B** Volcano plot of differentially expressed genes for conditions of interest

The workflow of this study is described in Fig. 1. DEGs were screened using the "limma" [16] package (R software) after merging GSE200828 and GSE108113 (11 control samples and 138 treatment samples) (Additional file 1: Table S1). A total of 574 DEGs were identified based on the FDR threshold < 0.05 and logFC ≥ 1.5. Among them, 228 genes were upregulated and 346 genes were downregulated (Fig. 2a, b) (Additional file 2: Table S2).

### Enrichment analysis

**Fig. 3:**
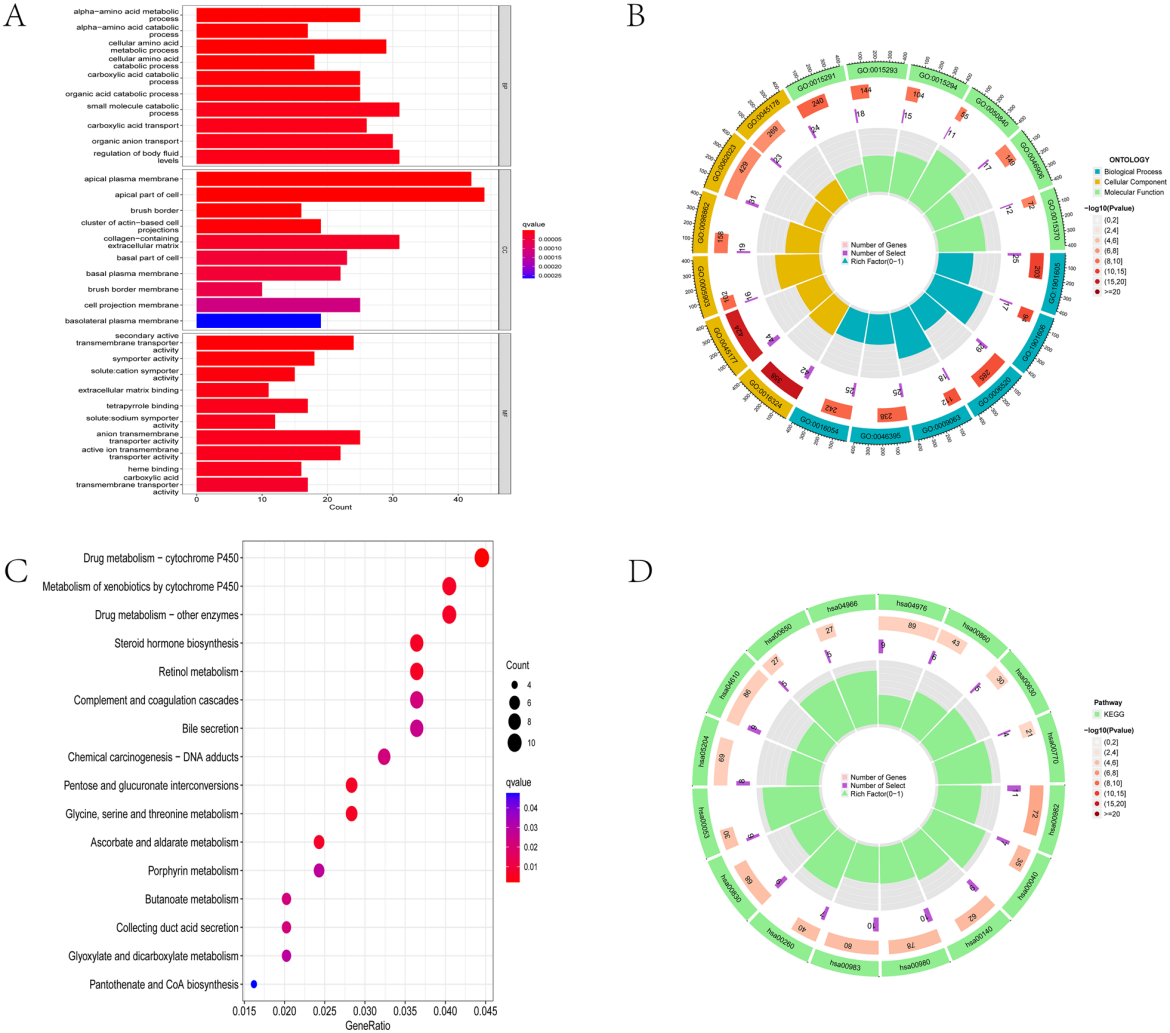
Enrichment analysis of the differentially expressed genes. **A**, **B** The GO enrichment analysis.** C**, **D** The KEGG enrichment analysis

We performed the GO and KEGG enrichment analyses to further understand the biological functions of DEGs and associated signaling pathways. In terms of biological processes, the GO enrichment analysis showed that the differentially expressed genes were mainly enriched in several terms, including “alpha-amino acid metabolic process” and “carboxylic acid catabolic process”. Regarding cellular components, they were mainly enriched in “apical plasma membrane” and “collagen-containing extracellular matrix”. As for molecular functions, they were mainly enriched in “anion transmembrane transporter activity” and "secondary active transmembrane transporter activity” (Fig. 3a, b) (Additional file 3: Table S3). The KEGG enrichment analysis showed that the DEGs were mainly enriched in metabolic and immune pathways, such as “metabolism of xenobiotics by cytochrome P450”, “complement” and “coagulation cascade” (Fig. 3c, d). (Additional file 4: Table S4).

### Gene set enrichment analysis (GSEA)

**Fig. 4:**
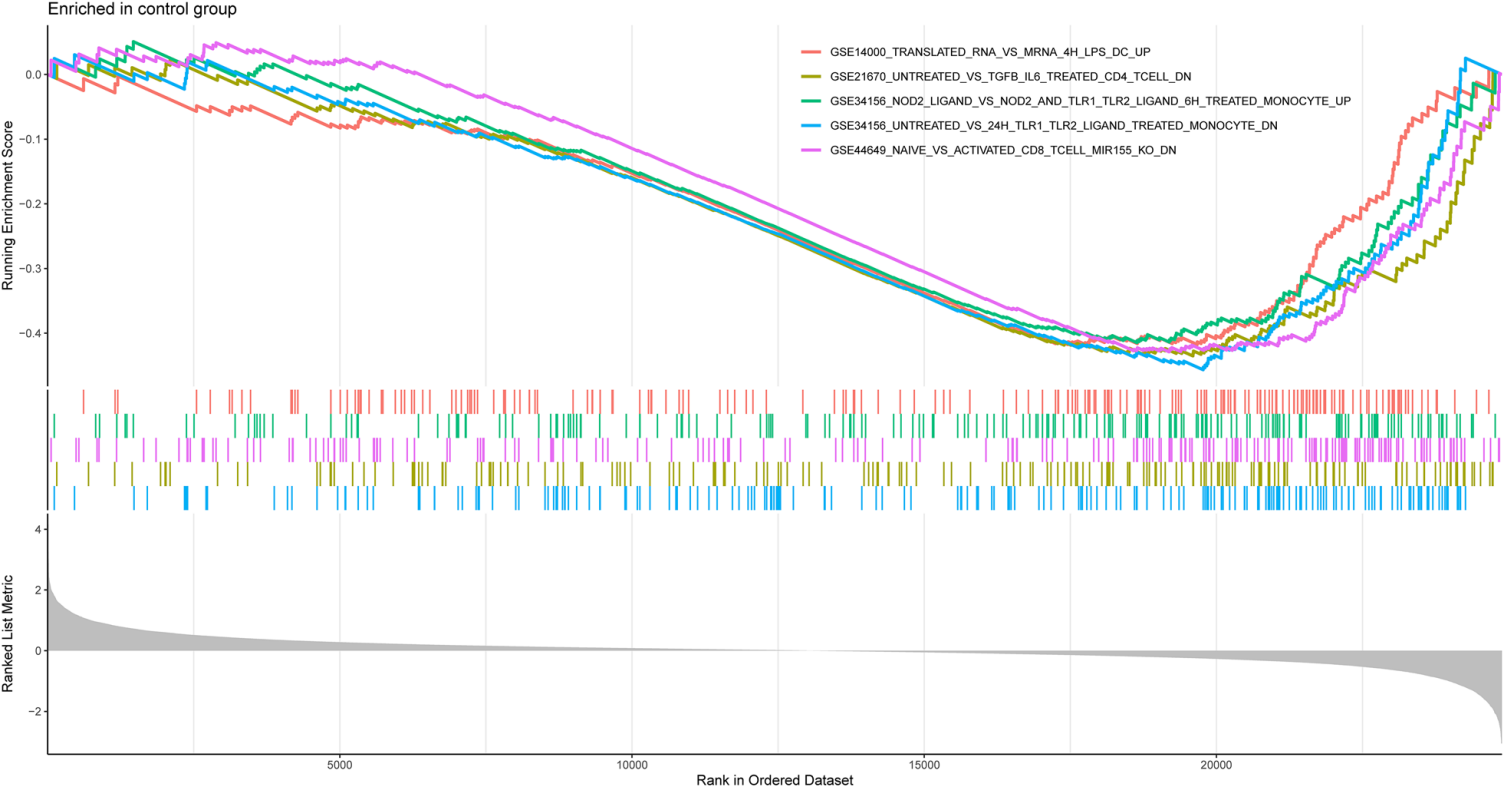
Enrichment plot of the immune-associated gene set analysis in control samples

**Fig. 5:**
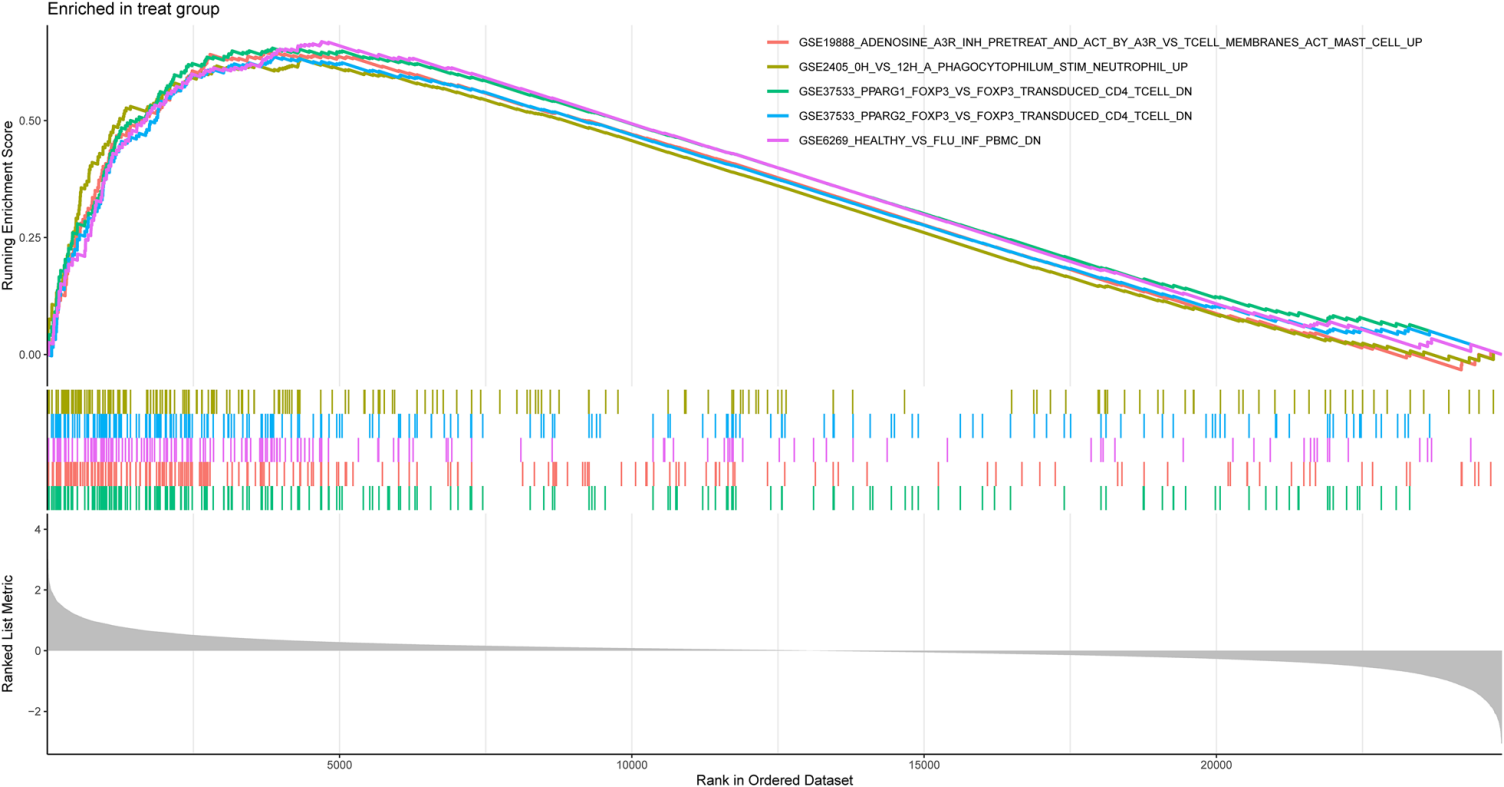
Enrichment plot of the immune-associated gene set analysis in MN samples

To further investigate the potential immune mechanisms in the pathogenesis of MN, we used the immune gene set from the MsigDB database as a reference. The obtained DEGs were subjected to the GSEA, with a normalized enrichment score (NES) > 1 and an adjusted *p*-value < 0.05. The top 5 significantly enriched gene sets were displayed in MN and control samples (Additional file 5: Table S5). According to the GSEA results, the significantly downregulated pathways in the control group include "GSE37533_PPARG1_FOXP3_VS_FOXP3_TRANSDUCED_CD4_TCELL" and "GSE6269_HEALTHY_VS_FLU_INF_PBMC" (*p*-value = 1.00E−10) (Fig. 4). The significantly elevated pathways in the experimental group include "GSE19888_ADENOSINE_A3R_INH_PRETREAT_AND_ACT_BY_A3R_VS_TCELL_MEMBRANES_ACT_MAST_CELL" and "GSE2405_0H_VS_12H_A_PHAGOCYTOPHILUM_STIM_NEUTROPHIL" (*p*-value = 1.00E−10) (Fig. 5). It can be inferred that, compared to the control group, the pathways of "GSE19888_ADENOSINE_A3R_INH_PRETREAT_AND_ACT_BY_A3R_VS_TCELL_MEMBRANES_ACT_MAST_CELL" and "GSE2405_0H_VS_12H_A_PHAGOCYTOPHILUM_STIM_NEUTROPHIL" are significantly activated, while the pathways of "GSE37533_PPARG1_FOXP3_VS_FOXP3_TRANSDUCED_CD4_TCELL" and "GSE6269_HEALTHY_VS_FLU_INF_PBMC" are suppressed in the experimental group. These findings imply that immune-related mechanisms may be crucial in the development of MN. Previous research has found that the number of T and B lymphocytes in the peripheral blood of patients with idiopathic membranous nephropathy is equivalent to that of healthy controls, but the CD4 +/CD8 + ratio is increased [8]. T cells also play a significant role in Heymann's nephritis, and reduction of CD4 + T cells eliminates IgG and C3 deposition and reduces proteinuria in rats, implying that CD4 + T cell-B cell interactions are critical in autoantibody synthesis [25]. Monocytes, as dendritic cells or progenitors of macrophages in tissues, play a key role in immune response regulation [26]. Jie According to Hou et al., elevated CD14 + CD163 + CD206 + M2 monocytes may be implicated in the pathogenesis of early IMN in adults and may serve as a sensitive biomarker for determining the severity of IMN [27]. A vaccination expressing CD40 DNA targeting dendritic cells was reported to prevent active Hyman's nephritis (HN) in experimental rats, an animal model of human autoimmune membranous nephropathy [28]. This shows that immune cell infiltration may be crucial in the development of membranous nephropathy. It warrants additional examination.

### Construction of the co-expression network

**Fig. 6:**
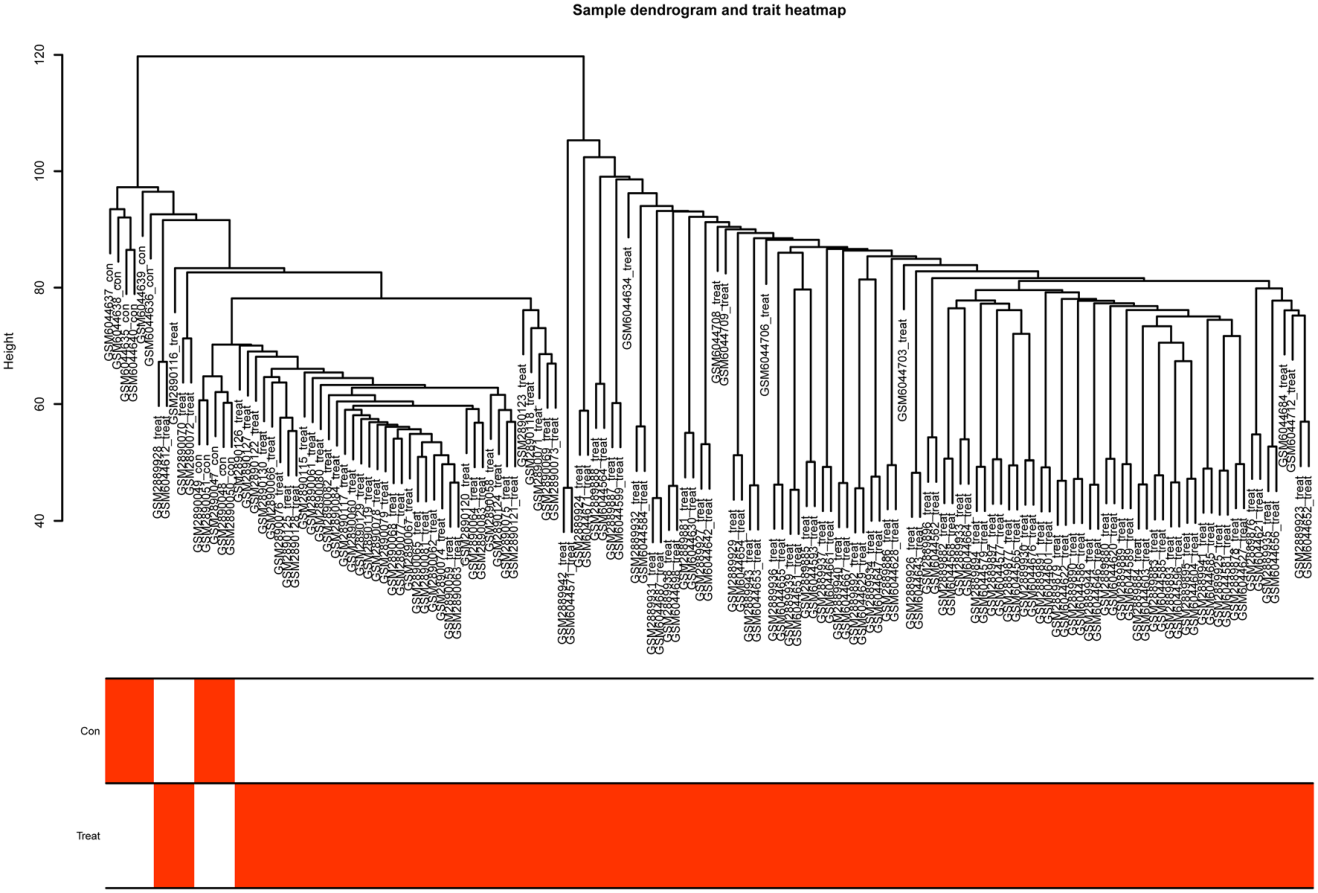
Sample dendrogram and trait heatmap. Con: control group, Treat: MN group

**Fig. 7:**
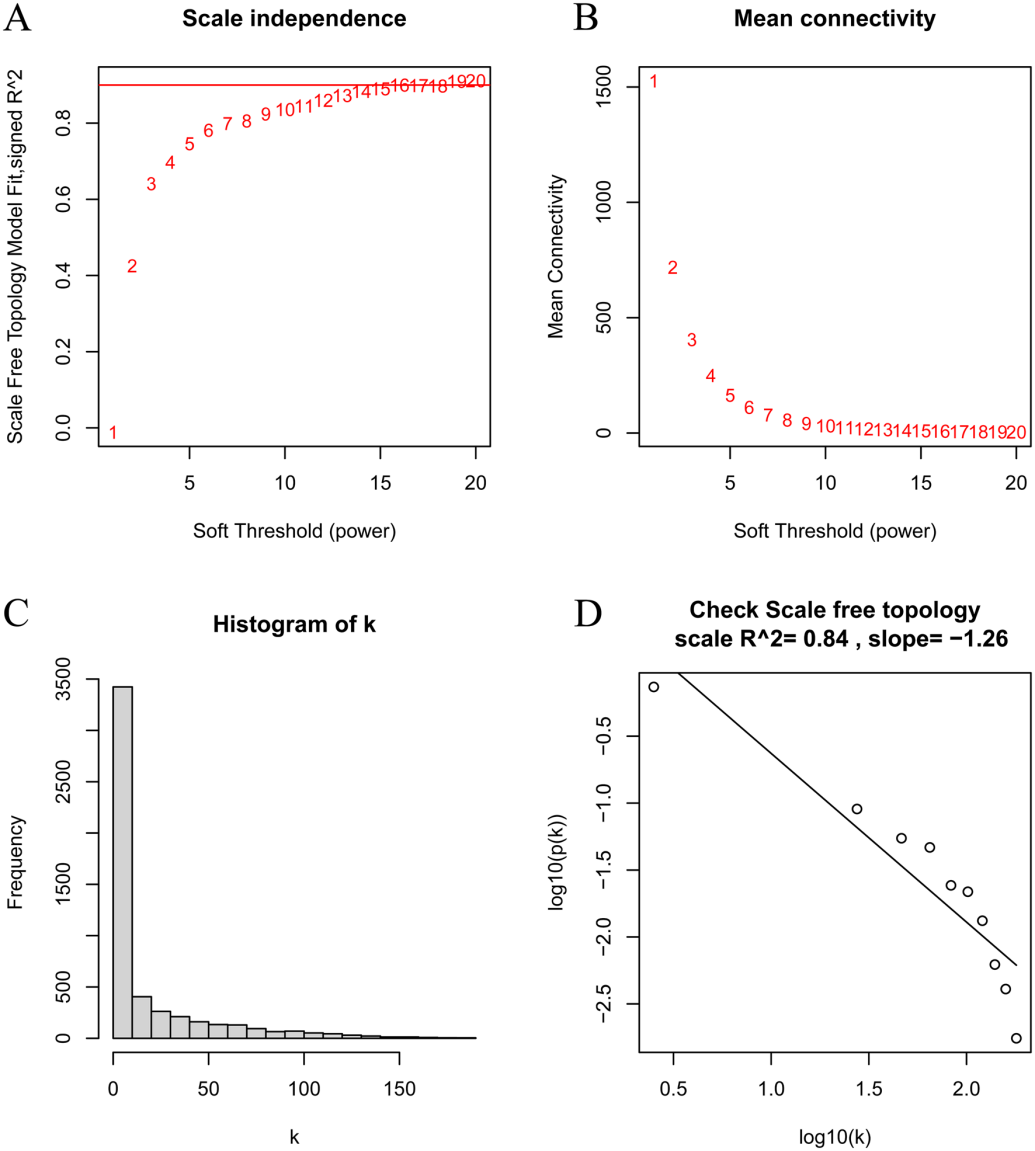
Determination of soft thresholds in WGCNA. **A**, **B** Analysis of scale-free fit indices and average connectivity for various soft threshold values of power. **C**, **D** Validation of scale-free topological networks at the power value of 12

**Fig. 8:**
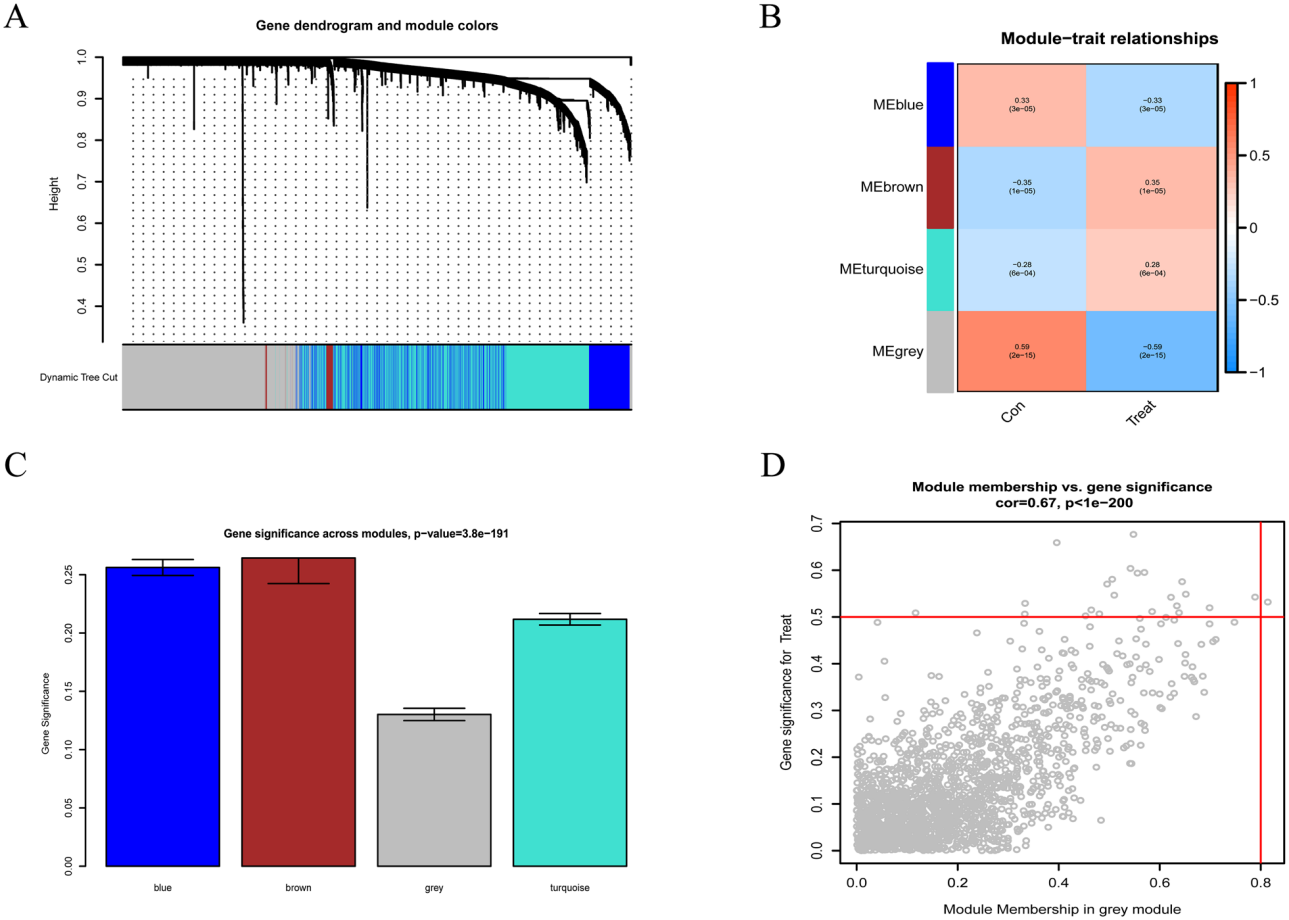
Construction of the WGCNA modules. **A** Sample hierarchical clustering tree diagram with different blocks representing the gene modules created by the dynamic tree cutting method.** B** Heatmap of module-clinical trait relationships. The grey modules were significantly associated with MN. **C** The gene significance scores are expressed in modules. **D** The scatter plot of members of the grey module with their gene significance

The WGCNA algorithm was used for the construction of the co-expression gene network. First, the samples were checked for missing values. Then, they were clustered to identify any outliers that included in our analysis (Fig. 6). The soft threshold was established by the soft concatenation function of WGCNA. When the power value was set to 12, the correlation coefficient was *R*^2^ = 0.84 with the slope = − 1.26, indicating that a satisfactory standard scale-free network could be constructed (Fig. 7). Finally, the modules with high similarity of feature genes were merged by the dynamic hybrid shearing method, obtaining four different color gene modules (Fig. 8a). The heat maps showed the correlation of the above modules between MN and control samples (Fig. 8b, c). Within the grey module, there was a good correlation between MM and GS (cor = 0.67, *p* < 1^e−200^) (Fig. 8d). We, therefore, selected the genes in the grey module for subsequent analysis.

### Identification of the hub genes

**Fig. 9:**
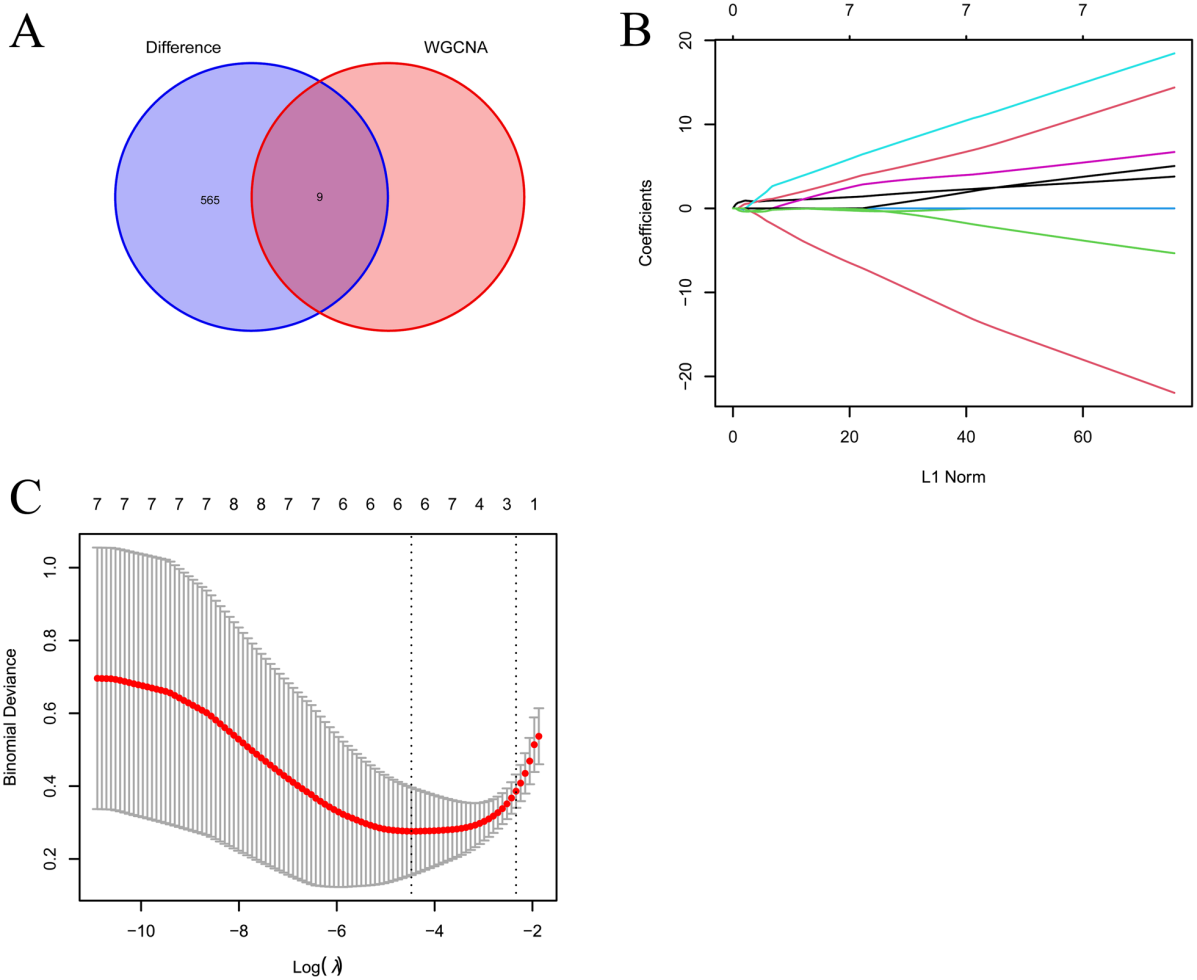
Screening of the hub genes. **A** Venn diagram of the intersection of differentially expressed genes and the grey modular genes. **B** LASSO regression analysis of the coefficient distribution maps for the hub genes.** C** The tenfold cross-validation obtaining the hub genes

Based on previous analysis, we obtained 9 candidate hub genes from the gray module. To further clarify the diagnostic value of the candidate hub genes, we obtained a total of 9 intersected genes (Fig. 9a). We subsequently performed the LASSO analysis to identify 6 hub genes: *ZYX*, *CD151*, *N4BP2L2-IT2*, *TAPBP*, *FRAS1* and *SCARNA9* (Fig. 9b, c). Previous studies have shown that ZYX, CD151, TAPBP and FRAS1 all appear to be associated with cell adhesion [29–32] and that two lncRNAs, N4BP2L2-IT2 and SCARNA9, play important roles in regulating human immune function [33, 34]. Their significance in membranous nephropathy, however, is unknown.

### The expression level of hub genes

**Fig. 10:**
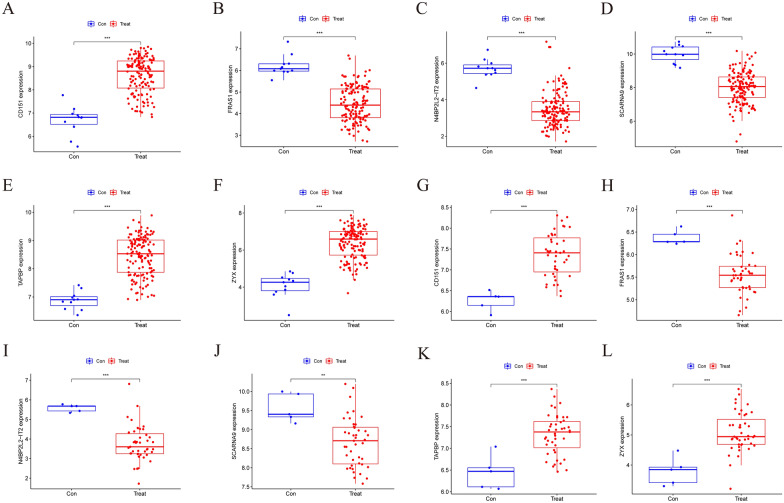
The expression level of the hub genes. **A**–**F** The expression level of the hub genes in the training datasets (GSE200828 and GSE108113).** G**–**L** The expression level of the hub genes in the validation dataset (GSE108112). Con: controls, Treat: MN group

**Fig. 11:**
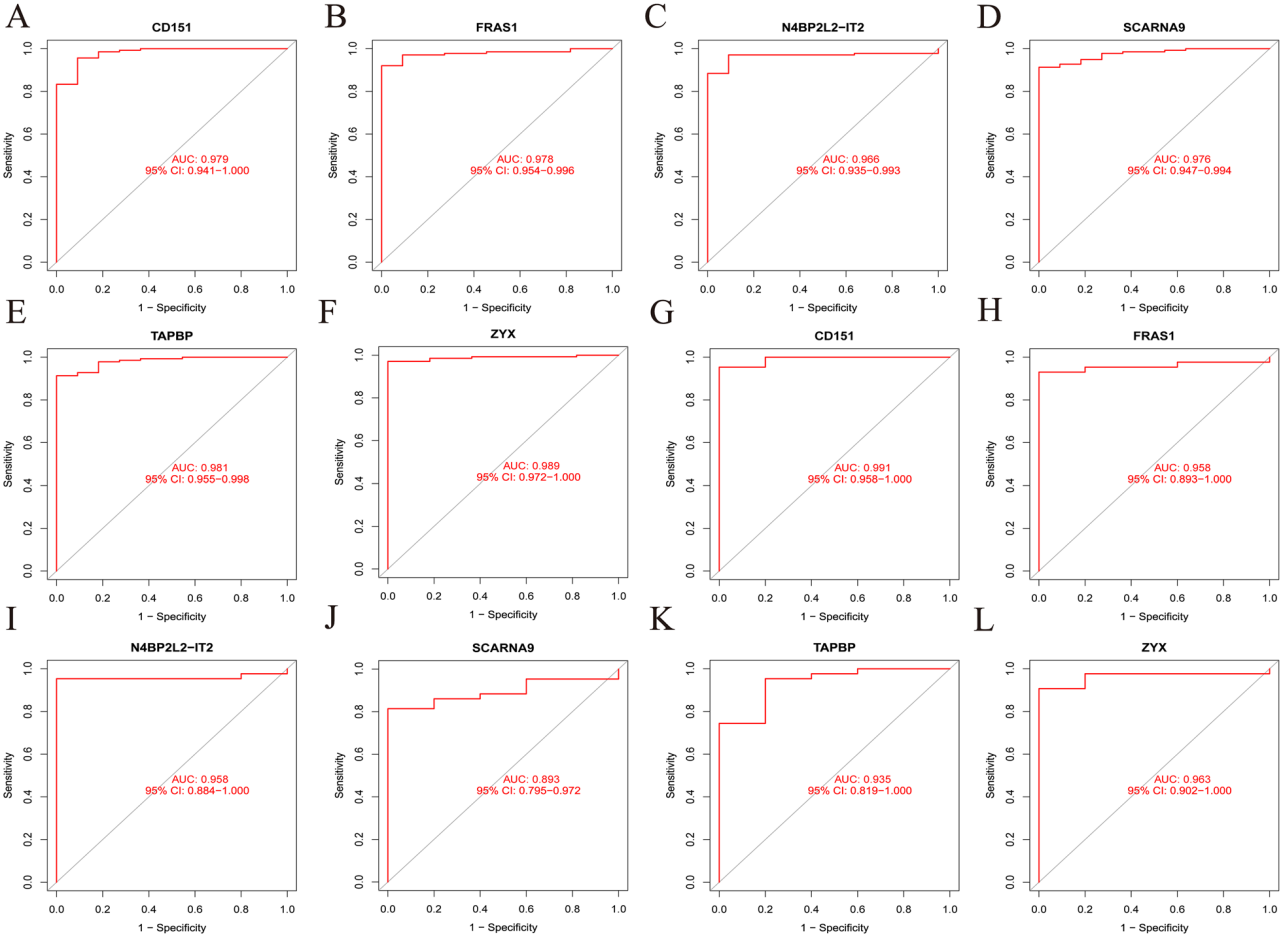
Validation of the diagnostic value of the hub genes. **A**–**F** Validation of the hub genes using the training datasets (GSE200828 and GSE108113). **G**–**L** Validation of the hub genes using the validation dataset (GSE108112). AUC, area under the curve

We designed box plots to observe the expression level of the 6 hub genes. The expression level of *CD151*, *TAPBP* and *ZYX* was significantly higher in MN samples than in normal samples (*p* < 0.001), whereas the expression level of *FRAS1*, *N4BP2L2-IT2* and *SCARNA9* was significantly lower in MN samples than in normal samples (*p* < 0.001) (Fig. 10a–f). The expression level of the hub genes was validated using the GSE108112 dataset, with the levels of *CD151*, *TAPBP*, *ZYX*, *FRAS1* and *N4BP2L2-IT2* being similar to those observed in the training set (Fig. 10g–l). The ROC curve was designed to evaluate the diagnostic value of the hub genes. The results showed that the AUC values of the hub genes were all > 0.95, suggesting a high diagnostic value (Fig. 11a–f). The accuracy of their clinical value was further validated using the GSE108112 dataset, showing that the AUC values of *ZYX*, *CD151*, *N4BP2L2-IT2*, *TAPBP* and *FRAS1* were > 0.9 while the AUC value of *SCARNA9* was 0.893 (Fig. 11g–l). Understanding the precise involvement of these important genes in the etiology of MN could have major clinical ramifications. For starters, these genes could be used as diagnostic biomarkers, assisting in the early detection of MN and risk assessment. Second, targeting these critical genes and their associated pathways could pave the door for new MN therapy options. Modifying their expression or activity may aid in the regulation of immune responses and the slowing of disease development. However, more study is required to evaluate these implications and investigate potential treatment strategies based on these findings. Overall, this study adds to our understanding of the molecular pathways underlying MN and has the potential to advance customized diagnosis and therapy approaches for MN patients.

### Immune cell infiltration

**Fig. 12:**
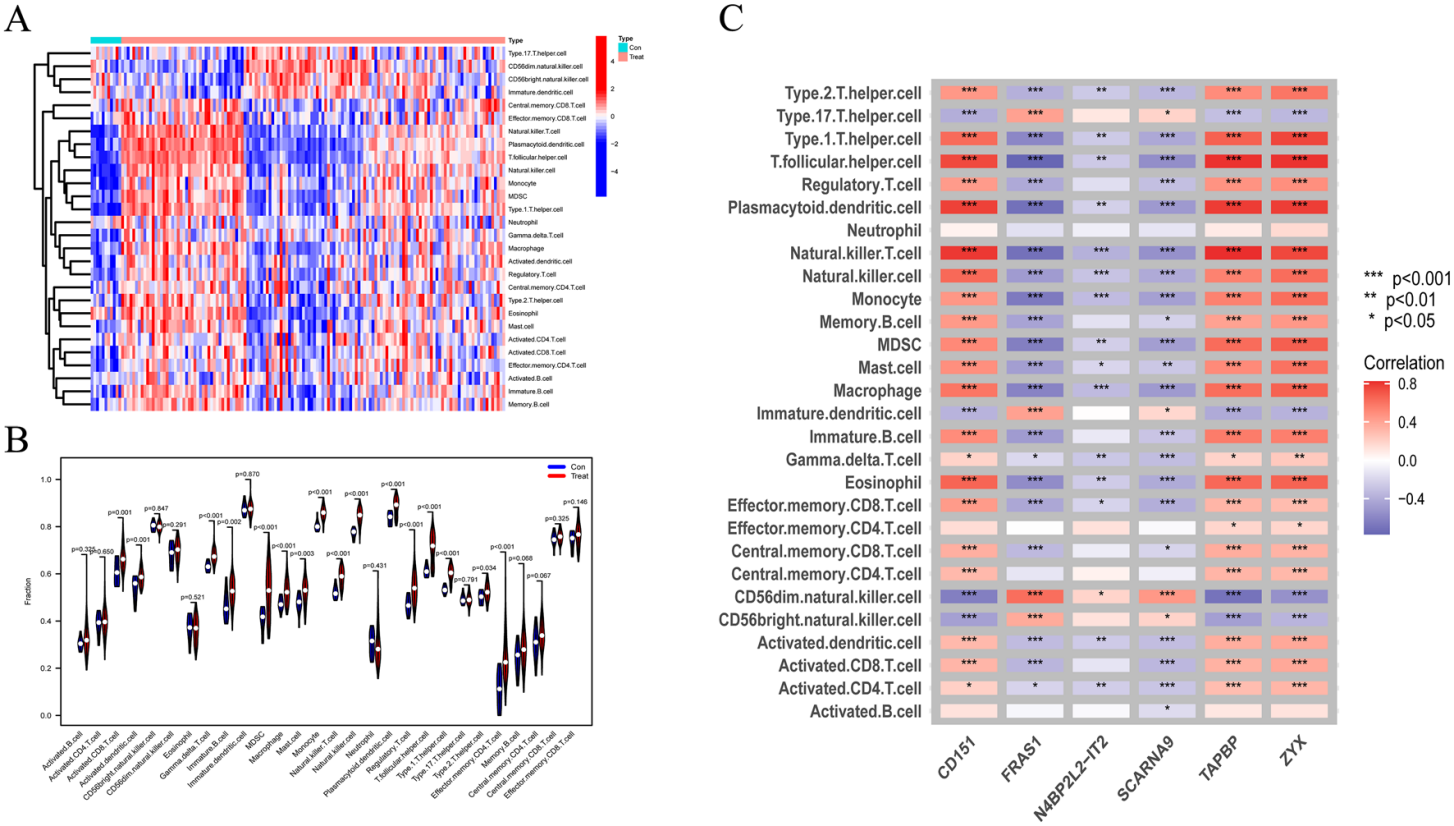
Infiltration of 28 immune cells in MN samples and correlation analysis with the hub genes. **A** Heatmap of different infiltrated immune cells. **B** Distribution of the 28 immune cells in MN and control samples.** C** Heatmap of correlation analysis between immune cells and hub genes. Con: controls, Treat: MN group

The immune cell infiltration status of healthy and MN samples is visualized in Additional file 6: Table S6. The results showed that CD8^+^ T cells, activated dendritic cells (aDCs), immature B cells, myeloid-derived suppressor cells (MDSC), macrophages, mast cells, monocytes, natural killer T cells, natural killer (NK) cells, plasmacytoid dendritic cells (pDCs), regulatory T cells, T follicular helper (Tfh) cells, T helper type 1 (Th1) cells, T helper type 2 (Th2) cells and CD4^+^ T cells were significantly more infiltrated in MN samples than in controls (Fig. 12a, b), suggesting that an abnormal immune function might play a role in the pathogenesis of MN. CD8 + T cells play a significant role in cell-mediated immune responses. CD8 + T lymphocytes in MN can detect and assault target cells that express specific antigens, resulting in inflammatory reactions and tissue damage [35]. Their cytotoxic activity may aggravate renal inflammation and injury, negatively altering glomeruli structure [36]. More study is needed to acquire a better understanding of the role of CD8 + T cells in the progression of MN, which could lead to new therapeutic approaches. Activated dendritic cells (aDCs) are antigen-presenting cells that are thought to play an important regulatory role in MN. T cells may be activated by aDCs that produce cytokines such as IL-12, IL-6, and TNF-. These cytokines may stimulate T cell proliferation and activation while also regulating inflammatory responses. ADCs' regulating function may be important in the immunopathogenesis of MN [37]. In-depth research into the functions of aDCs in MN may aid in understanding their unique involvement in disease pathophysiology, resulting in innovative intervention and treatment options. The correlation analysis of the characteristic genes with immune infiltrating cells showed that pDCs, Tfh cells, Th1 cells, Th2 cells and macrophages were positively correlated with *CD151*, *TAPBP* and *ZYX* (*p* < 0.001), whereas NK cells, monocytes and macrophages were negatively correlated with *FRAS1*, *SCARNA9* and *4BP2L2-IT2* (*p* < 0.001) (Fig. 12c). These findings suggested that specific immune cells might be involved in the development of MN.

## Discussion

Bioinformatics has been widely used in various fields of medical research with an important role in the diagnosis of diseases, the development of drugs and treatment personalization. By identifying novel biomarkers and key genes associated with membranous nephropathy (MN), it is important to improve disease diagnosis, facilitate early detection and improve prognostic prediction, thereby benefiting MN patients and potentially extending to other renal and immune-related diseases. The importance of the key genes identified in this study as potential therapeutic targets. The application of bioinformatics to the analysis of human patient data will enable the development of personalized treatment strategies to optimise therapeutic efficacy and reduce adverse effects. The application of bioinformatics to analyse high-throughput data and suggest gene clusters associated with a specific disorder has become an efficient research method [38, 39].

MN is distinguished by glomerular basement membrane thickening and significant podule fusion. MN is presently thought to be an autoimmune illness, with disease progression intimately tied to the inflammatory immunological process. Although numerous significant target antigens in MN, including as PLA2R, THSD7A, and Nell-1, have been discovered, the molecular processes underlying glomerular damage in MN remain unknown.

We identified putative important genes linked with MN in this study by combining MN gene expression profiles from the GEO database and using WGCNA and machine learning approaches. ssGSEA was used to analyze 28 immune cell subsets from MN patients, and their connection with diagnostic markers was explored. This study introduces novel concepts and strategies for the diagnosis and treatment of MN. We found that DEGs were mainly enriched in functions and pathways such as “cellular metabolism” and “immune response”. The pathogenesis of MN might be related to both aspects. Gao et al*.* found significant metabolic changes in MN patients, suggesting that such events may be related to the severity of MN [40]. Other studies showed that an abnormal activation of the complement system played an important role in the pathogenesis of MN. However, a recombinant humanized monoclonal antibody against the complement protein C5 (eculizumab) was not effective in the treatment of MN [41]. To further investigate the relationship between immune function and MN, we performed the GSEA using the immune marker gene sets from the MsigDB database to provide a reference. We found that CD4^+^ T cells, neutrophils, mast cells and peripheral blood mononuclear cells were significantly enriched in MN samples while monocytes, thymocytes, CD8^+^ T cells and DCs were significantly enriched in control samples, suggesting that the development of MN might be related to the abnormal activation of specific immune cells. Li et al. found a significant increase in myeloid-derived suppressor cells (MDSCs) in the peripheral blood, an increase in Th2 and Th17 immune responses and a positive correlation with disease activity in MN patients [42]. Consistently with our findings, MN patients reported a significantly lower number of regulatory T cells and an increased CD4^+^/CD8^+^ T cell ratio than healthy individuals [43]. In a retrospective cohort study, Tsai et al*.* found that some laboratory values were predictive of poor renal outcomes in patients with MN, including low serum levels of complement C3, high and intermediate granulocyte-lymphocyte ratios and a high platelet-lymphocyte ratio [44]. In accordance with our results, these studies suggested that the balance of immune cells plays a crucial role in the pathological progression of MN.

We used the WGCNA to screen out signature genes, with the candidate hub genes being identified by the LASSO algorithm (*ZYX*, *CD151*, *N4BP2L2-IT2*, *TAPBP*, *FRAS1* and *SCARNA9*). To investigate the diagnostic value of these genes, we used another separate dataset (GSE108112) for validation and plotting ROC curves. All six genes displayed a high diagnostic value: *CD151*, *TAPBP* and *ZYX* were highly expressed in MN samples whereas *FRAS1*, *N4BP2L2-IT2* and *SCARNA9* were scarcely expressed in MN samples. *ZYX* is a zinc-binding phosphoprotein concentrating at focal adhesions and along the actin cytoskeleton [45]. *ZYX* played an important role in DNA repair [46], apoptosis [47] and natural immunity [48]. *CD151* is a principal regulator of the laminin-binding integrins signaling process supporting the normal function of the glomerular filtration barrier [49]. Previous studies showed that *CD151* was closely associated with the formation of nephrogenic proteinuria [50]. A recent study described that a novel variant in *CD151* was associated with nephrogenic proteinuria and microscopic hematuria, reinforcing the importance of *CD151* in the pathogenesis of MN [31]. TAP-binding protein (*TAPBP*), also known as tapasin, is a transmembrane glycoprotein that played an important role in the processing and presentation of class I antigens [51]. Tapasin promoted antigen presentation and specific recognition of the MHC I peptide complex by CD8^+^ cytotoxic T lymphocytes, favoring the release of cytotoxic proteins. An increased expression of tapasin ameliorated the infiltration of CD8^+^ cytotoxic T lymphocytes into tumor cells leading to activation of anti-tumor immune responses [52]. *FRAS1* encodes an extracellular matrix protein located in the sublaminar region of the basement membrane that was associated with the development of a congenital kidney disease [53, 54]. Fraser extracellular matrix complex subunit 1 (*FRAS1*) genetic variant was significantly associated with the progression of chronic kidney disease to end-stage renal disease [55]. N4BPL2 intronic transcript 2 (*N4BP2L2-IT2*) is a long-stranded non-coding RNA actively operating during autophagy [56]. Previous studies found that *N4BP2L2-IT2* was involved in the development of MN [34]. Small Cajal body-specific RNA* 9* (*SCARNA9*) is an immune-related long non-coding RNA that influenced the development and progression of many tumors by regulating the immune components of the tumor microenvironment [57, 58]. However, studies concerning *SCARNA9* and MN have not been performed.

The diagnostic relevance of these hub genes in clinical practice could play a key role in early identification, illness prognosis, and individualized therapy methods for MN. These hub genes may serve as novel biomarkers for clinical testing if they are validated and developed further, giving doctors with more accurate and timely diagnostic information.

Furthermore, therapeutic strategies based on the functional roles of these hub genes might be investigated. For example, creating medications or therapy strategies that target ZYX, which is important in DNA repair, apoptosis, and natural immunity, could be effective in treating MN and related diseases. As potential therapeutic targets, CD151 and TAPBP could be examined for creating drugs that intervene in the progression and development of membranous nephropathy.

We used the ssGSEA algorithm to assess the extent of immune cell infiltration in MN and control samples. We found that the numbers of CD8^+^ T cells, aDCs, immature B cells, MDSC, macrophages, mast cells, monocytes, natural killer T cells, NK cells, pDCs, Th cells and CD4^+^ T cells were significantly higher in MN samples than in controls. The CD4^+^ T cells are responsible for coordinating the killer cells, the antibodies and the phagocytes that will eliminate the pathogens [59]. The Th1 cells mainly release cytokines such as IL-2, IFN-γ and TNF-β [60] while Th2 cells secrete IL-4, IL-5, IL-6 and IL-10 [59]. Previous studies have described a significant imbalance of Th cell subsets in patients with MN. Specifically, the Th2/Th1 cell ratio was increased, the expression of IL-4 was upregulated and the expression of IFN-γ was reduced, correlating with the severity of proteinuria [61]. The Th17/Treg imbalance was observed in a variety of autoimmune diseases [62]. A recent study found an association among increased Th17, thrombosis and recurrence in patients with MN [63]. The effectiveness of rituximab suggested an important role for B cells in patients with MN [64]. Current research focuses on the restoration of immune tolerance by Treg cells and manipulation of antigen expression [65, 66].

This study also has several limitations. First, the microarray dataset employed in this work has a limited sample size. Second, while we examined the enrichment of differential genes for activities and pathways, the specific processes that link them have yet to be thoroughly understood. More research with bigger sample sizes is required to find potential biomarkers linked to MN. Furthermore, experimental data are required to evaluate the functional roles of the identified MN genes.

## Conclusion

In summary, we identified six hub genes (*ZYX*, *CD151*, *N4BP2L2-IT2*, *TAPBP*, *FRAS1* and *SCARNA9*) associated with the pathogenesis of MN through an integrated bioinformatics analysis. We performed an immune infiltration analysis, providing further insight into the immune mechanisms of MN. Future studies will use a combination of large sample size and in vivo experiments to validate new diagnostic and therapeutic biomarkers for MN.

### Supplementary Information

**Additional file 1.** GSE108113 and GSE200828 merged data.

**Additional file 2.** The differentially expressed genes of merged data.

**Additional file 3.** GO enrichment analyses.

**Additional file 4.** KEGG enrichment analyses.

**Additional file 5.** Gene set enrichment analysis.

**Additional file 6.** The immune cell infiltration status of healthy and MN samples.

## Data Availability

The datasets analyzed for this study can be found in the Gene Expression Omnibus (GEO) database (http://www.ncbi.nlm.nih.gov/geo/). GSE200828 was downloaded from (https://www.ncbi.nlm.nih.gov/geo/query/acc.cgi?acc=GSE200828), GSE108113 was downloaded from (https://www.ncbi.nlm.nih.gov/geo/query/acc.cgi?acc=GSE108113). GSE108112 was downloaded from (https://www.ncbi.nlm.nih.gov/geo/query/acc.cgi?acc=GSE108112).

## Abbreviations

MN: Membranous nephropathy
DEGs: Differentially expressed genes
GSEA: Gene set enrichment analysis
WGCNA: Weighted gene co-expression network analysis
LASSO: Least absolute shrinkage and selection operator
ROC: Receiver operating characteristic
ssGSEA: Single-sample gene set enrichment analysis
ZYX: Zyxin
CD151: CD151 molecule
N4BP2L2-IT2: N4BPL2 intronic transcript 2
TAPBP: TAP-binding protein
FRAS1: Fraser extracellular matrix complex subunit 1
SCARNA9: Small Cajal body-specific RNA
